# A comparison of dose between cone beam computed tomography and conventional computed tomography in broad beam geometry

**DOI:** 10.1093/rpd/ncaf122

**Published:** 2026-03-13

**Authors:** Joel Larsson, Zakarias Grönqvist, Olivia Carlstein, Sara Asplund

**Affiliations:** Department of Medical Radiation Sciences, Institute of Clinical Sciences, Sahlgrenska Academy, University of Gothenburg, Gula stråket 2 B SU/Sahlgrenska, SE-413 45 Gothenburg, Region Västra Götaland, Sweden; Section of Diagnostic Imaging and Functional Medicine, NU Hospital Group, SE-461 85 Trollhättan, Region Västra Götaland, Sweden; Birger Sjöberggymnasiet, Kunskapsförbundet Väst, Idrottsgatan 3, SE-462 23 Vänersborg, Region Västra Götaland, Sweden; Section of Diagnostic Imaging and Functional Medicine, NU Hospital Group, SE-461 85 Trollhättan, Region Västra Götaland, Sweden; Section of Diagnostic Imaging and Functional Medicine, NU Hospital Group, SE-461 85 Trollhättan, Region Västra Götaland, Sweden

## Abstract

A unified computed tomography dose index (CTDI) which includes all scattered radiation was used to investigate the difference in absorbed dose to the patient between a computed tomography (CT) and a cone beam CT (CBCT). The unified CTDI, denoted CTDI*_300,w_*, was measured using a 100 mm pencil ionization chamber at three positions covering 300 mm at each of the five phantom slots in three CTDI body phantoms. The dose length product of a lumbar spine protocol for the modalities was comparable (CT: 153 mGycm, CBCT: 126 mGycm), though the normalized CTDI*_300,w_* for the CT was 4.6 times higher than for the CBCT. The comparison between CTDI*_300,w_* and the two dose indexes provided by the systems showed a substantial underestimation for both modalities. The most accurate, but also the most inconvenient method to compare absorbed dose is to use the CTDI*_300,w_*. Most important is to always use the same dose index.

## Introduction

Navigation by a cone beam computed tomography (CBCT) in orthopaedic surgery of the spine may have many benefits. For example, preoperative computed tomography (CT) examinations may be acquired directly on the operating room table avoiding alignment issues of the spine [1]. Furthermore, the possibility of acquiring a postoperative CT examination whilst the operative field is still sterile, may eliminate cases wherein the patient needs to revisit the operating room for repositioning of hardware [1]. Traditional methods using 2D fluoroscopy may give lower accuracy for the complex procedures such as the placement of pedicle screws in spinal surgery [2, 3]. Although operation time may sometimes be extended using a CBCT, the hospitalization stay for a patient may be shortened [3].

Traditional fluoroscopy devices such as c-arms use the metric dose-area product (DAP) to estimate the radiation exposure to the patient. The DAP gives the total radiation output from the device. Hence, the exposed area outside a patient is included and may result in an overestimation of the absorbed dose to a patient. Such overestimation is unlikely to be of concern in surgery of the spine using fluoroscopy devices, as the radiation field is seldom extended outside the body contours. However, a CT will almost always expose areas outside the patient. Hence the radiation exposure to the patient is estimated to a PMMA phantom through the computed tomography dose index (CTDI) [4, 5]. A CBCT can be used both as a c-arm and as a CT with a broad beam geometry. The original CTDI concept is not valid in geometries broader than 40 mm [6]. Thus, the radiation exposure from an examination on a CBCT is often expressed in DAP and/or cone beam dose index (CBDI). The CBDI describes the average absorbed dose within the length of the 100 mm pencil ionization chamber used in CTDI measurements and is intended to give a reasonable estimate of the absorbed dose to the phantom without considerations about the penumbra region [7]. Further, the dose index used in CT systems in broad beam geometries is often determined by the third edition of IEC 60601–2-44 (CTDI*_IEC3.0_*) [8]. However, the CTDI*_IEC3.0_* may substantially underestimate the absorbed dose to the phantom cylinder of PMMA, as the scattered radiation in the dose tails is not measured directly but scaled from a reference collimation [9]. Thus, a comparison of dose index between a CBCT and CT may not be straight forward, especially when the CBCT may collimate the x-ray field in two directions and alter the dose distribution in the CTDI phantom.

The study aims to investigate the difference in absorbed dose to a patient between a CBCT and a CT using a unified dose index, which includes the scattered radiation from the dose tails in the CTDI phantom.

## Materials and methods

The investigated CBCT was a Loop-X (medPhoton, Brainlab) and the CT was a Revolution Apex Power Pro (GE Healthcare). Both systems are used clinically at the NU Hospital Group. The CBDI*_w_* and CTDI*_IEC3.0,w_* were used to estimate the radiation exposure from examinations by the CBCT and the CT manufacturer, respectively. The present study investigated the difference between CTDI*_IEC3.0,w_* and a unified dose index CTDI*_300,w_* described below. All measurements were performed using a 100 mm RTI CT pencil ionization chamber together with a Piranha 657 and a RTI chamber adapter. The whole system was calibrated at radiation quality RQT 9 one week before the first measurements. The expanded uncertainty of the absorbed dose readings at reference conditions is ±2.0%.

### CTDI_*IEC3.0,w*_

For the CT, the CTDI*_IEC3.0,w_* was used for collimations greater than 40 mm. The CTDI*_IEC3.0,w_* was based on the traditional CTDI*_100_* which was calculated as follows,


1 \begin{eqnarray*} {CTDI}_{100}={\int}_{-50\ mm}^{50\ mm}\frac{D_{ref}(z)}{col{.}_{ref}} dz, \end{eqnarray*}

where the reference dose integrated along z (D*_ref_*(z)) was normalized for a reference collimation (col.*_ref_*). The CTDI*_100_* was scaled by the ratio between free-in-air measurements to calculate the CTDI*_IEC3.0,100_* according to the following:


2 \begin{eqnarray*} {CTDI}_{IEC\mathrm{3.0,100}}={CTDI}_{100}\bullet \frac{CTDI_{free\ air, col{.}_{new}}}{CTDI_{free\ air, col{.}_{ref}}}, \end{eqnarray*}

where CTDI*_free air,col.new_* and CTDI*_free air,col.ref_* were the free-in-air measurements for the new and reference collimation used in equation (1), respectively. The free-in-air measurement of each collimation was the sum of multiple measurements obtained by a 100 mm pencil ionization chamber placed to cover the collimation width in the z-direction, which has been recommended by others [10]. The CTDI*_IEC3.0,w_* was calculated according to IEC 60601–2-44 as follows,


3 \begin{eqnarray*} {CTDI}_{IEC3.0,w}=&\;\frac{1}{3}{CTDI}_{IEC\mathrm{3.0,100}, centre} \nonumber\\ &+\,\frac{2}{3}{CTDI}_{IEC\mathrm{3.0,100}, peripheral}, \end{eqnarray*}

where CTDI*_IEC3.0,100,centre_* and CTDI*_IEC3.0,100,peripheral_* were calculated from CTDI*_IEC3.0,100_* for the centre position and peripheral positions in the CTDI phantom, respectively. The CTDI*_IEC3.0,100,peripheral_* was calculated as an average of the four measurements at the peripheral positions.

### A unified dose index, CTDI_*300,w*_

**Figure 1:**
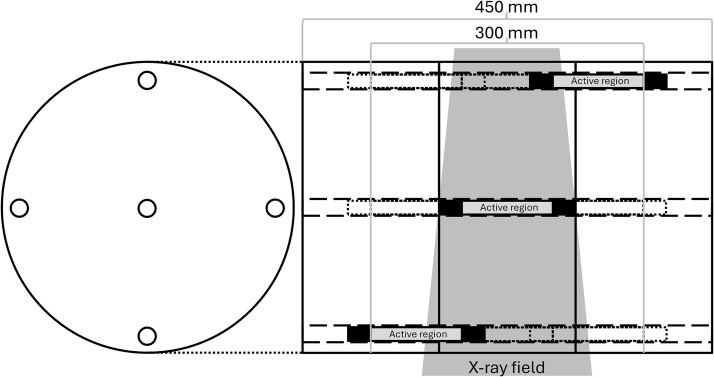
Schematic views of the three CTDI phantoms (total length, 450 mm) used for measurement of CTDI*_3BP,w_*, where the active region of the pencil ionization chamber was placed in three positions at each of the five phantom slots to cover a measurement length of 300 mm.

The unified dose index used in this investigation was inspired by the report of AAPM task group 111: the future of CT dosimetry [11] and was measured using three CTDI body phantoms placed in a line to a total length of 450 mm (length of each phantom was 150 mm, Fig. 1). The 100 mm pencil ionization chamber was placed at three positions in the z-direction at each of the five phantom slots (the central and the four peripheral). The ion chamber was placed against a jig in the form of a rod to assure the position of the measurements. The rod was marked at the three lengths such that the placement uncertainty was no more than a couple of millimetres. The absorbed dose at each chamber position was measured for separate scans, such that the active region covered a length of 300 mm (Fig. 1). The measured absorbed dose was summed up at each phantom slot and used to calculate a weighted CTDI for the active region according to the traditional CTDI formalism [4]. Thus, the unified dose index is further on denoted as CTDI*_300,w_*.

### Comparison of CTDI_*300,w*_ between CBCT and CT

The measured CTDI*_300,w_* was normalized by the tube current (mA) and exposure time (s) and compared between the modalities. Further, the different dose indexes provided by the systems for CBCT (CBDI*_w_*) and the CT (CTDI*_IEC3.0,w_*) were also normalized and compared. This to investigate the possible overestimation by comparing the dose indexes straight off. For the CBCT, the CTDI*_300,w_* was measured at one default collimation (150 × 150 mm^2^) used for lumbar spine examinations. The default scan mode acquired a scan in 29.3 s using a half rotation geometry, where the tube was moving across the lower half of the phantom. The tube current was manually set to 5 mA. For the CT, the CTDI*_300,w_* was measured at seven collimations (5, 40, 80, 100, 120, 140, and 160 mm) using a rotation time of 1 s with a tube current of 600 mA. However, the normalized dose indexes (nCTDI*_300,w_*, nCBDI*_w_*, and nCTDI*_IEC3.0,w_*), were compared between the modalities at an interpolated collimation of 150 mm to match the collimation of the CBCT. The tube potential used for both modalities was 120 kV.

### CTDI_*300,w*_ compared to CBDI_*w*_ and CTDI_*IEC3.0,w*_

The relative difference between nCTDI*_300,w_* and the normalized dose indexes provided by the systems were analyzed to investigate a possible underestimation by the CBDI*_w_* for the CBCT and by CTDI*_IEC3.0,w_* for the CT (nCTDI*_300,w_*/nDose index, i.e. nCTDI*_300,w_*/nCBDI*_w_*, and nCTDI*_300,w_*/nCTDI*_IEC3.0,w_*). For the CT, the comparison was made for the seven collimations to analyze how the estimated nCTDI*_300,w_* followed the nCTDI*_IEC3.0,w_* provided by the system and for the CBCT the comparison was made for the default collimation. The effect of the longer phantom length for the nCTDI*_300,w_* was investigated by a ratio between a normalized CTDI*_100,w_* (nCTDI*_100,w_*, traditional CTDI formalism, phantom length of 150 mm) and a normalized CTDI*_100,w_* obtained from measurements at the middle positions (z-direction) of the three CTDI body phantoms (nCTDI*_100,3BP,w_*, phantom length of 450 mm). The CTDI*_100,w_* was based on the CTDI*_100_* measurements for the CTDI*_IEC3.0,w_* and CTDI*_100,3BP,w_* on the measurements for the CTDI*_300,w_*. The difference between the measured and the provided values was only estimated for the CT using the CTDI*_IEC3.0,w_*. In the present study, the reference collimation used in the calculation of the measured CTDI*_IEC3.0,w_* was 40 mm.

### Comparison of clinical protocols

In the present study, clinical protocols for lumbar spine examinations at the NU Hospital Group were used to measure and compare the dose length product (DLP) between the modalities; two CT protocols (a standard dose and a low dose protocol) and one default CBCT protocol. The DLP values for these clinical protocols were based on the CTDI*_300,w_*. A scanning region of 150 mm over the CTDI body phantom was used to estimate the tube current output by the localizer radiographs for both modalities. The nCTDI*_300,w_* was each used to calculate the DLP. The clinically used collimations in this comparison were 80 mm for the CT protocols and the default collimation of 150 × 150 mm^2^ for the CBCT protocol.

## Results

### Comparison of CTDI_*300,w*_ between CBCT and CT

The nCTDI*_300,w_* measured for the CBCT (collimation, 150 x 150 mm^2^) and the CT (collimation, 150 mm) was 0.0184 mGy/mAs and 0.0842 mGy/mAs, respectively (Table 1). Thus, the nCTDI*_300,w_* showed to be 4.6 times higher for the collimation used by the CT than the collimation in two directions used by the CBCT. In contrast, the normalized dose indexes provided by the systems for these collimations were 0.0110 mGy/mAs, and 0.0644 mGy/mAs for CBCT (nCBDI*_w_*), and CT (nCTDI*_IEC3.0,w_*), respectively (Table 1). A comparison between these provided dose indexes showed to be 5.9 times higher for the CT. Hence, the difference in dose index between the modalities was overestimated by 28% by those provided by the systems.

**Table 1:** Dose indexes normalized by the tube charge (noted with n) for each nominal collimation. The nDose index is based on the values provided by the systems and refers to nCTDI_*IEC3.0,w*_ and nCBDI_*w*_ for the CT and CBCT, respectively. nCTDI_*100,w*_ and nCTDI_*100,3BP,w*_ are the normalized CTDI_*100,w*_ measured using a phantom length of 150 mm (traditional formalism) and a phantom length of 450 mm (in the middle of three phantoms), respectively. nCTDI_*IEC3.0,w*_ and nCTDI_*100,w*_ were only measured for the CT system. The dose readings had an uncertainty of ±2.0%.

Modality	Collimation (mm)	nDose index^a^ (mGy/mAs)	nCTDI*_IEC3.0,w_*^b^ (mGy/mAs)	nCTDI*_300,w_*^b^ (mGy/mAs)	nCTDI*_100,w_*^b^ (mGy/mAs)	nCTDI*_100,3BP,w_*^b^ (mGy/mAs)
CT	5	n.a.	n.a.	0.1487	0.1063	0.1110
CT	40	0.0684^c^	0.0669	0.0930	0.0668	0.0691
CT	80	0.0678^c^	0.0669	0.0896	0.0624	0.0644
CT	100	0.0671^c^	0.0656	0.0878	0.0596	0.0616
CT	120	0.0658^c^	0.0643	0.0865	0.0563	0.0583
CT	140	0.0644^c^	0.0632	0.0853	0.0518	0.0540
CT	150^d^	0.0644^c^^,^^d^	0.0631^d^	0.0842^d^	0.0505^d^	0.0516^d^
CT	160	0.0644^c^	0.0630	0.0831	0.0492	0.0492
CBCT	150 × 150	0.0110^e^	–	0.0184	–	0.0096

^a^Normalized dose index based on values provided by the system.

^b^Normalized dose index based on values measured in the present study.

^c^CTDI*_IEC3.0,w_.*

^d^Linear interpolated value between 140 and 160 mm.

^e^CBDI_w_.

### CTDI_*300,w*_ compared to CBDI_*w*_ and CTDI_*IEC3.0,w*_

The nCTDI*_300,w_* for the CT were shown to be ~60% higher at the 5 mm collimation in comparison to the other investigated collimations (Table 1). The measured nCTDI*_300,w_* differed by 2% to the nCTDI*_300,w_* provided by the system (Table 2). Further, the nCTDI*_300,w_* was in general 32% (range 29%–36%, Table 2) higher for all investigated collimations compared to the nCTDI*_300,w_* provided by the system. For the CBCT, the nCTDI*_300,w_* was 67% higher than the nCBDI*_w_* provided by the system (Table 2). The nCTDI*_100,w_* was consistently ~4% lower than the nCTDI*_100,3BP,w_* for all investigated collimations for the CT (Table 2). Further, the nCTDI*_100,3BP,w_* was ~75% of the nCTDI*_300,w_* at collimations up to 40 mm and above 40 mm the ratio between the dose indexes gradually declined down to 59% (Table 2). For the CBCT, the nCTDI*_100,3BP,w_* was approximately half of the nCTDI*_300,w_*.

**Table 2:** Ratios between investigated dose indexes normalized by the tube charge (noted with n) for each nominal collimation. nCTDI*_*IEC3.0,w*_*^a^/ nCTDI*_*IEC3.0,w*_*^b^ refers to the ratio between the normalized CTDI value provided by the system and the value measured in the present study. nCTDI_*300,w*_ is the normalized measured unified dose index defined in the present study. The nDose index is based on the dose indexes provided by the systems and refers to nCTDI_*IEC3.0,w*_ and nCBDI_*w*_ for the CT and CBCT, respectively. nCTDI_*100,w*_ and nCTDI_*100,3BP,w*_ are the normalized CTDI_*100,w*_ measured using a phantom length of 150 mm (traditional formalism) and a phantom length of 450 mm (in the middle of three phantoms), respectively. The dose readings had an uncertainty of ±2.0%.

Modality	Collimation (mm)	nCTDI*_IEC3.0,w_*^a^/ nCTDI*_IEC3.0,w_*^b^	nCTDI_300*,w*_^b^/ nDose index^a^	nCTDI*_100,w_*^b^/ nCTDI*_300,w_*^b^	nCTDI*_100,3BP,w_*^b^/ nCTDI*_300,w_*^b^	nCTDI*_100,w_*^b^/ nCTDI*_100,3BP,w_*^b^
CT	5	n.a.	n.a.	0.71	0.75	0.96
CT	40	1.02	1.36^c^	0.72	0.74	0.97
CT	80	1.01	1.32^c^	0.70	0.72	0.97
CT	100	1.02	1.31^c^	0.68	0.70	0.97
CT	120	1.02	1.32^c^	0.65	0.67	0.96
CT	140	1.02	1.33^c^	0.61	0.63	0.96
CT	150^d^	1.02^d^	1.31^c^^,^^d^	0.59^d^	0.61^d^	0.96^d^
CT	160	1.02	1.29^c^	0.56	0.59	0.95
CBCT	150 × 150	–	1.67^e^	–	0.52	–

^a^Normalized dose index based on values provided by the system.

^b^Normalized dose index based on values measured in the present study.

^c^nCTDI_300*,w*_/nCTDI*_IEC3.0,w_.*

^d^Linear interpolated value between 140 and 160 mm.

^e^nCTDI_300*,w*_/nCBDI_*w*_.

### Comparison of clinical protocols

The estimated DLP of a lumbar spine examination over a region of 150 mm was 126 mGycm for the CBCT using the default protocol and 153 mGycm for the CT using the standard-dose protocol. The difference in DLP between the modalities was 22% and thus less than the difference in nCTDI*_300,w_*. Further, the DLP for a low-dose protocol over the same region was 44 mGycm, i.e. ~30% of the DLP for the standard-dose protocol.

## Discussion

In the present study, the unified dose index CTDI*_300,w_* was measured to investigate the difference in absorbed dose to a patient between a CBCT and a CT. These modalities use different dose indexes to describe the radiation exposure from an examination. The CBCT uses the CBDI_w_ which is not intended to account for scattered radiation in the penumbra region [7]. However, for the CT in the present study, the CTDI*_IEC3.0_* is used to estimate the absorbed dose to the phantom by including the dose contribution of the scattered radiation by a scaling factor based on a reference collimation [12]. Hence, a comparison between these two different dose indexes may overestimate the difference in absorbed dose between modalities considerably, which has been shown in the present study.

The consistent underestimation of the absorbed dose from scattered radiation by the CTDI*_IEC3.0_* compared to the CTDI*_300,w_* shown in the present study indicates that CTDI*_IEC3.0_* may be used to compare the relative absorbed dose for CT systems. However, a CBCT may collimate the x-ray field around the patient (x/y-direction) such that the phantom is partial irradiated, which may alter the dose distribution within the CTDI phantom compared to a fully irradiated phantom. It is possible that these two dose distributions will not propagate with the same factor as the collimation is increased in the z-direction. Hence, a limitation of the study is that the CTDI*_300,w_* was only investigated for the default collimation for the CBCT, as the size of the beam could only be steplessly changed, making it difficult to reproduce the exact collimation repeatedly. A future investigation may test the dose indexes at the end point positions of the collimation in the x/y-direction to analyze the effect of not exposing the whole CTDI phantom.

The positioning of the pencil ionization chamber may be a critical factor to errors in the determination of the CTDI*_300,w_*. However, the jig constructed to repeat the measurements at consistent positions helped to reduce the uncertainty to a max of a couple of millimetres, which in total will be in the range of the internal uncertainty of the detector system of ±2%. Further, a systematic uncertainty in collimation size may also contribute to the total uncertainty. In the present study the uncertainty would be in the same range as the positioning uncertainty (±2%) for every one-millimetre deviation in collimation.

The CTDI*_w_* (denoted CTDI*_100,w_* in the present study) is defined in a CTDI phantom, where the absorbed dose is measured using a 100 mm ionization chamber and the dose profile in such phantom is usually wider than the phantom itself for any clinical collimation [9]. The present study indicates that the nCTDI*_100,w_* consistently underestimates the contribution of the scattered radiation by ~4% due to the insufficient length of the CTDI body phantom. Consequently, the length of the ionization chamber will not be long enough to detect all the scattered radiation. According to the present study, a 100 mm ionization chamber will consistently measure ~75% of the total radiation from collimations up to 40 mm. Hence, this indicates the traditional CTDI formalism to be valid for relative measurements in this range of collimations, which has been proposed by others [6]. This is further consistent with a previous simulation study which has shown that the difference in CTDI ratios between collimations is ˂2% in this collimation range [13]. Furthermore, the relative low variation of the nCTDI*_300,w_* between collimations from 40 mm to 160 mm indicates that three CTDI body phantoms and a 300 mm measure length were sufficient to estimate a dose index that capture the scattered radiation for the investigated collimations. These indications are in accordance with other reported results which has shown underestimations of the relative dose to be ˂5% for ionization chambers of 300 mm and phantoms of 450 mm [14]. The substantially underestimation of the nCTDI*_100,w_* to nCTDI*_300,w_* by 56% for a 160 mm collimation is also consistent with previously reported results from Monto Carlo simulations [15].

Although the difference in nCTDI*_300,w_* between the investigated modalities was over four times higher for the CT, the difference in DLP used at the clinic was less than a quarter. Hence, it is possible that the benefits for a patient undergoing CBCT examinations during orthopaedic lumbar surgery may not be dependent on absorbed dose to the patient, as long as the examined area does not exceed that of a clinical CT examination. The advantages of using a CBCT may instead be prioritized, so that the surgical procedure if necessary can be adjusted directly in the operating room based on higher image quality than 2D methods [1]. However, the image quality obtained by a low-dose protocol for CT has been proposed to be sufficient to be used for pre- and postoperative controls, although low-dose CT has been graded to generate a less “sharp reproduction of the cortical and the trabecular bone” than radiography [16, 17]. Thus, a dose reduction for the default protocol of the CBCT may be possible and should be subject for further investigation to optimize the absorbed dose to a patient during lumbar spine surgery.

The CTDI*_300,w_* was the most accurate dose index investigated in the present study, as it included all scattered radiation to determine the absorbed dose to the CTDI phantoms. However, the three CTDI phantoms made the measuring of this index the most inconvenient method. Methods requiring only one CTDI phantom may probably be preferred to estimate the difference in absorbed dose between these modalities. The CTDI*_IEC3.0,w_* would have been a good candidate for such purpose. However, the difficulties in adjusting the collimation of the CBCT system to a valid known size for the reference measurements makes this alternative less attractive. Amongst the dose indexes studied, the CBDI*_w_* might be the most pragmatic index to use when comparing absorbed dose from modalities due to its simplicity. This, of course, implies that the CBDI*_w_* must be measured for the CT system before such a relative comparison can be made. Further, if a more accurate estimate of the absorbed dose to a patient is required, the results from the present study may be used. Although only the default collimation was investigated for the CBCT, a correction by 1.67 (ratio of CTDI*_300,w_* and CBDI*_w_*) could give a rough estimate. In the authors’ experience, collimation is rarely changed much from the default size for lumbar spine examinations.

## Conclusions

The DLP based on the CTDI*_300,w_* for a standard-dose-lumbar-spine examination was comparable between a CBCT and a CT for the same scan length, though the nCTDI*_300,w_* showed to be considerably higher for the CT. The investigation also underlines a substantial underestimation of the absorbed dose estimated by the CBDI*_w_* and CTDI*_IEC3.0,w_*. The most accurate, but also the most inconvenient method to compare absorbed dose between modalities is to use the CTDI*_300,w_* formalism. However, most important is to always use the same type of dose index.
